# *Candida auris* Identification and Rapid Antifungal Susceptibility Testing Against Echinocandins by MALDI-TOF MS

**DOI:** 10.3389/fcimb.2019.00020

**Published:** 2019-02-18

**Authors:** Mansoureh Vatanshenassan, Teun Boekhout, Jacques F. Meis, Judith Berman, Anuradha Chowdhary, Ronen Ben-Ami, Katrin Sparbier, Markus Kostrzewa

**Affiliations:** ^1^Bruker Daltonik GmbH, Bremen, Germany; ^2^Institute for Biodiversity and Ecosystem Dynamics, University of Amsterdam, Amsterdam, Netherlands; ^3^Westerdijk Fungal Biodiversity Institute, Basidiomycete and Yeast Research, Utrecht, Netherlands; ^4^Department of Medical Microbiology and Infectious Diseases, Canisius Wilhelmina Hospital, Nijmegen, Netherlands; ^5^Centre of Expertise in Mycology Radboudumc, CWZ, Nijmegen, Netherlands; ^6^Department of Molecular Microbiology and Biotechnology, George S. Wise Faculty of Life Sciences, Tel Aviv University, Tel Aviv, Israel; ^7^Department of Medical Mycology, Vallabhbhai Patel Chest Institute, University of Delhi, New Delhi, India; ^8^Infectious Diseases Unit, Tel Aviv Sourasky Medical Center, Tel Aviv, Israel; ^9^Sackler Faculty of Medicine, Tel Aviv University, Tel Aviv, Israel

**Keywords:** *C. auris*, MALDI-TOF MS, Identification, rapid antifungal susceptibility testing, echinocandins

## Abstract

*Candida auris* was first reported in an ear swab from Japan in 2009; it then promptly spread over five continents and turned into a global nosocomial problem. The main challenges faced by many researchers are the *mis*-identification by conventional methods in clinical laboratories and failure in treatment. About 90% of *C. auris* strains are intrinsically resistant to fluconazole (FLU), and it is developing resistance to multiple classes of available antifungals. Echinocandins are the most potent class of antifungals against *C. auris*; however, reduced susceptibility to one or many echinocandin drugs has been recently observed. Thus, the main issues addressed in this paper are the fast and accurate identification of *C. auris* derived from Sabouraud dextrose agar and blood culture bottles as well as the rapid antifungal susceptibility test by MALDI-TOF MS. This study successfully identified all isolates of *C. auris* (*n* = 50) by MALDI-TOF MS, with an average log score of ≥ 2. An accuracy of 100% was found on both agar plate and blood culture bottles. MALDI Biotyper antibiotic susceptibility test-rapid assay (MBT ASTRA) was used for rapid antifungal susceptibility testing (AFST). A comparison between MBT ASTRA and the Clinical and Laboratory Standards Institute guidelines (CLSI) detected a sensitivity and specificity of 100% and 98% for anidulafungin, and 100% and 95.5% for micafungin, respectively. A categorical agreement of 98% and 96% was calculated for the two methods. For caspofungin, sensitivity and specificity of 100 and 73% were found, respectively, with a categorical agreement of 82%. MBT ASTRA has the great potential to detect *C. auris* isolates non-susceptible against echinocandin antifungals within 6 h, which makes it a promising candidate for AFST in clinical laboratories in the future.

## Introduction

*Candida auris* is a recently-emerged *Candida* species first isolated from human samples of the external ear in Japan in 2009 (Satoh et al., 2009), and had spread to more than 30 countries in < 10 years (Saris et al., 2018). *C. auris* is primarily detected in patients with a long period of hospitalization in intensive care units (ICU). It causes diseases ranging from superficial skin infections to invasive bloodstream infections (BSI) with high mortality rates (30% to 60%) (Vincent et al., 2009; Chowdhary et al., 2016, 2017; Prakash et al., 2016; Cortegiani et al., 2018; Ruiz-Gaitán et al., 2018). In some hospitals in Asia *C. auris* is the second most common isolated species from blood cultures (Mathur et al., 2018). *C. auris* is resistant to fluconazole (FLU), and is also regularly reported as a multidrug resistant (MDR) yeast (Lee et al., 2011; Cortegiani et al., 2018; Meis and Chowdhary, 2018). Previous published studies showed increased *C. auris* MICs to all three major antifungal classes (Shin et al., 2012; Arendrup and Patterson, 2017; Lockhart et al., 2017; Chowdhary et al., 2018; Cortegiani et al., 2018). Echinocandins are the most effective drugs against *C. auris* infections (Sears and Schwartz, 2017; Jeffery-Smith et al., 2018). The majority of studies have proposed a tentative MIC breakpoint (in μg/mL) for resistance i.e., ≥ 4 for anidulafungin and micafungin, and a MIC breakpoint ≥ 2 for caspofungin (Ben-Ami et al., 2017; Spivak and Hanson, 2018; Tsay et al., 2018). In addition, echinocandin-resistant *C. auris* isolates have been observed on rare occasions in different geographical areas (Chowdhary et al., 2018). Notably, 36% of echinocandin resistant *Candida* strains have cross-resistance to azole antifungals (Arendrup and Patterson, 2017; Arendrup et al., 2017b; Chowdhary et al., 2018). Thus, knowing the degree of echinocandin resistance in specific *C. auris* isolates is critical for choosing appropriate antifungal drug therapeutic strategies. Considering the MDR propensity of this yeast the treatment of *C. auris* remains a challenge as also its identification in the routine microbiology laboratories (Ben-Ami et al., 2017; Spivak and Hanson, 2018). *C. auris* has been misidentified as *C. haemulonii, C. famata*, and even *S. cerevisiae* by conventional biochemical techniques and some commercial methods (Chowdhary et al., 2013; Ceyssens et al., 2017; Spivak and Hanson, 2018). Several studies have reported misidentification of *C. auris* as *C. haemulonii* by VITEK 2, if the database is not updated (Girard et al., 2016; Ben-Ami et al., 2017; Khan et al., 2018). Molecular methods remain the most reliable and accurate available approaches, although they are expensive and not used routinely in clinical laboratories (Ben-Ami et al., 2017; Spivak and Hanson, 2018; Tsay et al., 2018). Matrix-assisted laser desorption ionization–time of flight mass spectrometry (MALDI-TOF MS) has recently been considered as a convenient, rapid and high throughput technology in the identification of variant microorganisms at the species level (Cendejas-Bueno et al., 2012; Croxatto et al., 2012; Kolecka et al., 2016). Some studies showed that MALDI-TOF MS can identify *C. auris* correctly and rapidly, differentiating it from other related species (Ghosh et al., 2015; Kathuria et al., 2015; Prakash et al., 2016; Schelenz et al., 2016). However, the specificity and sensitivity of MALDI-TOF MS to a collection of *C. auris* isolates has not been tested. Taken together, identification and antifungal susceptibility tests for *C. auris* infection remain a challenge in the clinical microbiology setting.

Here, we investigated the MALDI-TOF MS based identification of *C. auris*. In addition, since a new trend to apply MALDI-TOF MS for AFST has appeared, Vella et al. (2013) have developed a rapid new AFST assay based on MALDI-TOF MS (Vella et al., 2017). They analyzed changes in the MS profile spectra induced by antifungals after 3 h of incubation (Vella et al., 2013). Although this method successfully worked for *C. albicans* isolates resistant to caspofungin (Vella et al., 2013), it did not accurately work for *C. glabrata* strains resistant to anidulafungin with known *FKS2* mutations (Vella et al., 2017). Therefore, more recently, MALDI Biotyper antibiotic susceptibility test- rapid assay (MBT ASTRA) was introduced for the rapid detection of *C. albicans* and *C. glabrata* strains resistant against caspofungin (Vatanshenassan et al., 2018), where resistant strains were detected within 6 h with a high sensitivity and specificity for both species. This method is a semi-quantitative phenotypic assay based on the comparison of cell growth in the presence of an antifungal to growth in a control setup without antifungal drug. The present research explores, for the first time, the capability of the MBT ASTRA to detect non-susceptible *C. auris* against the echinocandin drug class, and the degree to which they agree with results from CLSI microdilution assays.

## Materials and Methods

### *Candida auris* Isolates

The isolates used in this study were obtained from either strain collections or stock cultures of routine samples for which no written informed consent is required. A total of 50 *C. auris* isolates derived from either strain collections (10 from the Centers for Disease Control and Prevention, United States and 23 isolates from the Westerdijk Institute, The Netherlands) or stock cultures of routine samples (17 isolates) of different geographical areas (India, Israel, South Africa) were analyzed (Supplementary Table 1). All isolates had already been identified as *C. auris* by different commercial and molecular techniques. The isolates were stored at −80°C and fresh overnight cultures on Sabouraud dextrose agar (SDA) were used for identification and antifungal susceptibility tests (AFST). The reference strains *C. parapsilosis* ATCC 22019 and *C. krusei* (*Pichia kudriavzevii*) ATCC 6258, were used as quality control strains for susceptibility testing by the CLSI microdilution method. Two *C. auris* strains, CBS 12372 (KCTC 17809) and CBS 10913 (DSM 21092), were used as quality control strains for echinocandin susceptibility testing by the CLSI method and the MBT ASTRA (Arendrup et al., 2017a; Ruiz-Gaitán et al., 2018).

### MALDI-TOF MS Species Identification

For the MALDI-TOF MS-based identification of *C. auris* the Bruker MBT Compass Library, Revision E MBT 7854 MSP Library was employed. Samples derived from agar plate were prepared according to the MALDI Biotyper standard protocol (Sauer et al., 2008; Clark et al., 2013; Vlek et al., 2014).

For species identification from blood cultures, blood culture bottles (BD Bactec Plus Aerobic/F; Becton Dickinson, Heidelberg, Germany) were enriched with 10 ml whole sheep blood and spiked with the respective *C. auris* strains. The bottles were incubated in a Bactec automated blood culture instrument (Becton Dickinson, Heidelberg, Germany) until they were positive for each isolate. Subsequently, the positive blood cultures were purified by MALDI Sepsityper kit (Bruker Daltonik GmbH, Germany) according to the manufacturer's recommendations (Clark et al., 2013; Lange et al., 2014; Vlek et al., 2014).

### CLSI Microbroth Susceptibility Testing

The MICs for anidulafungin (Pfizer, New York, United States), micafungin (Astellas, Toyama, Japan) and caspofungin (Sigma-Aldrich, Germany) were determined in duplicate by the CLSI standard microdilution method according to guideline M60 (November 2017) (Clinical Laboratory Standards Institute. Performance Standards for Antifungal Susceptibility Testing of Yeasts; approved, 2017). Slow-growing strains that could not be visually evaluated after 24 h, were analyzed after 48 h incubation at 37°C. So far, respective breakpoints for susceptibility/resistance classification have not been defined by CLSI and are suggested in this study.

### MBT ASTRA

The *in vitro* antifungal susceptibility test was carried out according to the standard MBT ASTRA method, as recently described (Vatanshenassan et al., 2018), using 2 fold serial dilutions of anidulafungin (ranging from 0.125 to 8 μg/ml), micafungin (ranging from 0.5 to 32 μg/ml), and caspofungin (ranging from 0.125 to 4 μg/ml) and an additional control without an antifungal. Briefly, supplemented RPMI 1640 medium (Sigma-Aldrich, Germany) and cell inoculum were prepared according to the EUCAST reference method (Arendrup et al., 2017a; Vatanshenassan et al., 2018). Incubation was performed at 37°C in a Thermo Mixer (Eppendorf, Germany) under agitation at 300 rpm for 6 h. Multi-well filter plates (1ml well, 0.45μm GHP, PALL, United States) were used to collect the cells after incubation by centrifugation at 4,000 × g for 5 min (Eppendorf, Germany). Next, the cells were rinsed twice with 200 μl sterile deionized water and once with 100 μl 75% ethanol. Cell lysis was performed by 10.5 μl 70% formic acid (Merck, Germany) and 10.5 μl 100% acetonitrile (Roth, Germany) directly on the filter and was repeated once again. For MALDI-TOF MS measurements, 1 μl lysate of each set up was spotted in duplicate onto a polished steel target plate (Bruker Daltonik, Germany) and overlaid with 1 μl MALDI matrix (10 mg/ml of α -cyano-4-hydroxy-cinnamic acid [α-HCCA] in 50% acetonitrile−2.5% trifluoroacetic acid; Bruker Daltonik, Germany) containing the MBT ASTRA Standard II (Bruker Daltonik, Germany). MALDI-TOF MS spectra were acquired and analyzed by MBT ASTRA prototype software (Cendejas-Bueno et al., 2012; Croxatto et al., 2012; Vatanshenassan et al., 2018).

### MBT ASTRA From Positive Blood Cultures

Twenty isolates from the strains described above were randomly selected; susceptible (*n* = 14) and non- susceptible (*n* = 6) strains to anidulafungin and micafungin, susceptible (*n* = 13), or non- susceptible (*n* = 7) to caspofungin. Preparation of blood cultures, incubation, and purification of positive cultures by MALDI Sepsityper kit was performed exactly as described above for strain identification. The pellet derived from the Sepsityper kit was re-suspended in 1 ml RPMI 1640 medium and used to prepare cell suspension of McF 0.5. All following steps of MBT ASTRA were performed as described above (Vatanshenassan et al., 2018).

### Data Analysis

MBT ASTRA prototype software written in “R” was employed for spectra analysis according to the procedure described by Lange et al. (Gibb and Strimmer, 2012; Lange et al., 2014; Vatanshenassan et al., 2018). First, the area under the curve (AUC) was calculated which is directly corresponding to the growth of the strain within the respective setup. Subsequently, the relative growth was calculated as a measure of the growth of the respective strain in the presence of antifungal: *RG* = (AUC_RPMI+antifungal_)/(AUC_RPMI_). The relative growth cut-off was set to 0.7 RG units for all experiments. Strains with an RG value above this threshold were considered as non-susceptible; strain with an RG similar or below 0.7 were considered as susceptible. The CLSI microdilution was considered as standard method for evaluation of MBT ASTRA. Since this study was only a proof-of-principle-study, the number of tested strains was limited for applying a specific statistical calculation.

## Results

### Strain Identification

All 50 strains of *C. auris* grown on SDA were correctly identified based on the MALDI Biotyper. The results, as shown in Table 1, indicate that 47 strains were identified with log (score) values of ≥ 2 and three strains with log (score) values ranging between 1.7 and 2. Likewise, identification from positive blood culture bottles detected 46 strains with high log (score) values ≥ 2 and only 4 strains with log (score) values ranging between 1.7 and 2. An absolute accuracy of 100% was calculated for *C. auris* identification using SDA and blood culture bottles (Table 1).

**Table 1:** Identification of 50 *C. auris* isolates derived from SDA plate agar and positive blood cultures by MALDI-TOF MS, respectively.

**Strains/culture medium**	**Number of strains**	**No of log (score) values**			**No of correct identified samples (%)**	**Accuracy**
		**≥2**	**<2 and ≥1.7**	**≤1.7**		
*C. auris*/Plate agar (SDA)	50	47	3	–	50 (100)	100%
*C. auris*/positive blood culture (BC)	50	46	4	–	50 (100)	100%

### CLSI Antifungal Susceptibility Test

The CLSI microdilution method was considered as the gold standard to evaluate the MICs obtained by the MBT ASTRA. Since the CLSI has not yet determined the breakpoints for *C. auris* against echinocandins, the MICs found in this study based on CLSI microdilution method, were considered as a reference to evaluate breakpoints obtained by MBT ASTRA. Accordingly, strains were divided into 44 susceptible and 6 non-susceptible strains against anidulafungin and micafungin. All six isolates harbored an S639F mutation in FKS1 gene as published previously (Chowdhary et al., 2018). Applying the CLSI method for caspofungin divided strains into 33 susceptible and 17 non-susceptible isolates (Table 2). All 6 non-susceptible strains confirmed by sequencing, were correctly detected by CLSI method. As shown in Table 3, the MICs based on CLSI microdilution were defined as follows: R > 4 μg/ml and S ≤ 4 μg/ml for anidulafungin, R > 8 μg/ml and S ≤ 8 μg/ml for micafungin, and R ≥ 1 μg/ml and S < 1 μg/ml for caspofungin, respectively (Table 3) (Supplementary Table 1).

**Table 2:** *In vitro* echinocandin class drugs susceptibility test using CLSI microdilution and MBT ASTRA for 50 *C. auris* isolates.

**Methods**	**Total no (Susceptible/Resistant)**		
	**Anidulafungin**	**Micafungin**	**Caspofungin**
CLSI	50 (44/6)	50 (44/6)	50 (33/17)
MBT ASTRA	50 (43/7)	50 (42/8)	50 (24/26)

**Table 3:** Comparison of suggested breakpoints against echinocandin class drugs for *C. auris* tested by CLSI microdilution and MBT ASTRA.

**Methods**	**MIC μg/ml**		
	**Anidulafungin**	**Micafungin**	**Caspofungin**
CLSI	R> 4 μg/ml S ≤ 4 μg/ml	R> 8 μg/ml S ≤ 8 μg/ml	R≥ 1 μg/ml S < 1 μg/ml
MBT ASTRA (SDA)	R> 4 μg/ml S ≤ 4 μg/ml	R> 8 μg/ml S ≤ 8 μg/ml	R≥ 2 μg/ml S < 2 μg/ml
MBT ASTRA (blood culture bottles)	R> 1 μg/ml S ≤ 1 μg/ml	R> 4 μg/ml S ≤ 4 μg/ml	R≥ 1 μg/ml S < 1 μg/ml

### *In vitro* Susceptibility Test by MBT ASTRA

**Figure 1:**
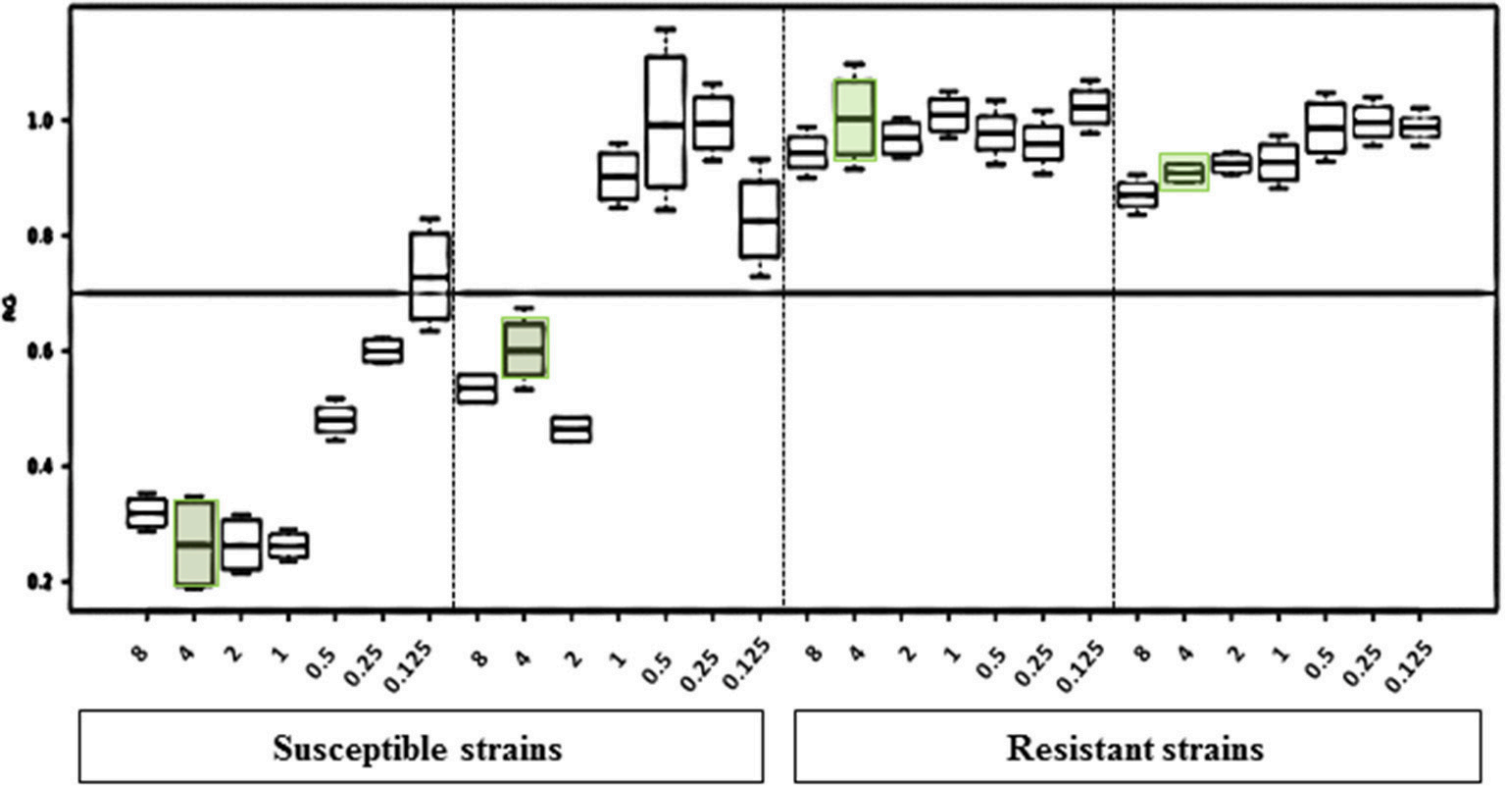
Results of the MBT ASTRA prototype software evaluation. The relative growth values of 2-fold serial dilutions of anidulafungin of 2 susceptible and 2 resistant *C. auris* strains after 6 h incubation show distinct differences. The susceptibility/resistance threshold was set at a RG value of 0.7. For susceptible strains, at the concentration of 4 μg/ml anidulafungin a significant reduction of the relative growth was observed.

**Figure 2:**
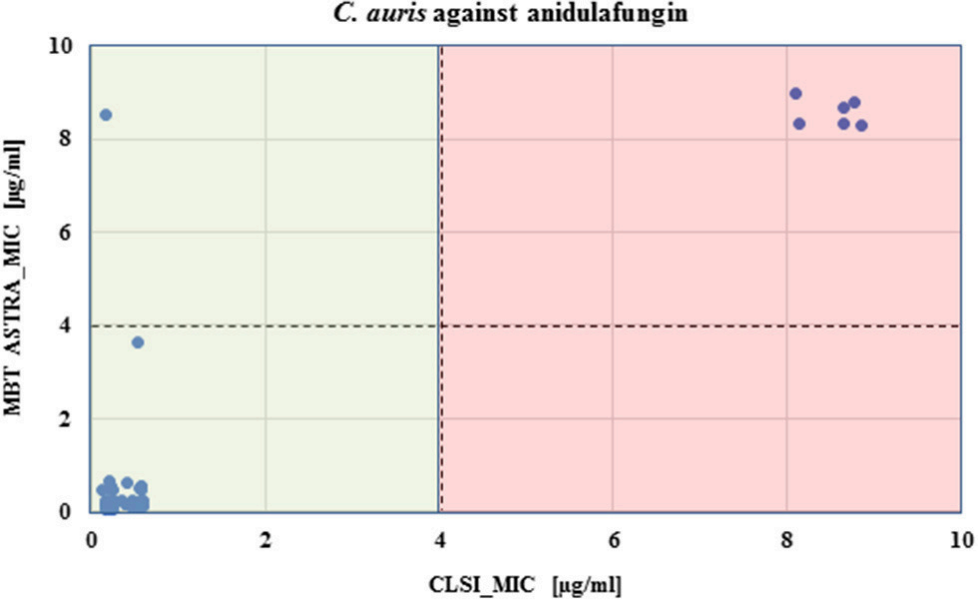
Comparison of CLSI microdilution MICs and MBT ASTRA MICs for *C. auris* isolates (*n* = 50) against anidulafungin. For each isolate, the MIC value derived by MBT ASTRA (*y* axis) was plotted against the MIC obtained by microdilution (x axis) for anidulafungin. The colored boxes indicate the MIC ranges according to CLSI; green, susceptible and red, resistant. The horizontal dashed line indicates the suggested cutoff defined for MBT ASTRA MIC, and the vertical dashed line shows the suggested cutoff determined for CLSI. A high agreement was observed between both approaches, and only one isolate susceptible against anidulafungin was wrongly detected by MBT ASTRA.

MBT ASTRA was performed for 50 strains as described above, and the area under the curve (AUC) was calculated by MBT ASTRA prototype software, based on the acquired spectra according to the growth of each strain at each antifungal concentration (Gibb and Strimmer, 2012). Further analysis determined that the minimal antifungal concentrations resulting in an RG value similar to or below the cut-off value of 0.7 were considered as the breakpoint for the respective strain. The RG values for four test strains (2 susceptible and 2 non-susceptible) are shown in Figure 1. For anidulafungin, the AUCs of the non-susceptible isolates were the same in absence and presence of the antifungal resulting in RGs close to 1; while for the susceptible strains, the AUCs were remarkably decreased in the presence of the drug resulting in decreasing RG values with increasing antifungal concentrations. The RG values of 42 susceptible strains already decreased at a concentration of ≤ 2 μg/ml of anidulafungin and for only one susceptible strain at a concentration of 4 μg/ml. An example of the respective boxplot is given in Figure 1. Next, the MICs derived from MBT ASTRA were determined, and subsequently compared to the MICs derived from CLSI microdilution (Figure 2) (Table 3). Accordingly, 4 μg/mL was defined as the anidulafungin breakpoint concentration. Findings indicated that 49 of 50 strains were classified correctly, with only one susceptible strain *mis*-classified by MBT ASTRA.

For micafungin, susceptible strains had a growth reduction only at higher concentrations of micafungin compared to anidulafungin. For micafungin at a concentration of 8 μg/mL, 42 susceptible strains were below the cut-off of 0.7. In contrast, all non-susceptible strains constantly grew above the cut-off of 0.7 for all concentrations up to 32 μg/ml. Table 3 shows that micafungin MBT ASTRA-MICs were in high agreement with the MICs derived from CLSI microdilution. Accordingly, there is a clear separation of susceptible and non-susceptible strains for micafungin with a breakpoint concentration of 8 μg/ml for both the CLSI reference method and MBT ASTRA. In addition, analysis of caspofungin susceptibility of the isolates found that the RGs of susceptible isolates were decreased at concentrations of 2 μg/ml, while the growth for non-susceptible strains at this concentration was in the same range that had been observed for the controls for all caspofungin concentrations. The breakpoint for the MBT ASTRA was one serial dilution higher than the separating MIC for the CLSI method (Table 3).

Overall, analysis of all 50 tested isolates by MBT ASTRA indicated a high agreement between anidulafungin and micafungin. One susceptible isolate was *mis*-labeled by both antifungals, but all 6 strains determined as being non-susceptible by microdilution were correctly categorized by MBT ASTRA (Table 2). For anidulafungin, a high agreement of 98%, and sensitivity and specificity of 100% and 98% were calculated, respectively. For micafungin, categorical agreement was 96%, and sensitivity and specificity were 100% and 95.5%, respectively (Table 4). In contrast, for caspofungin twenty-four susceptible and 26 non-susceptible isolates were detected by MBT ASTRA (Table 2). All 17 strains classified as non-susceptible by the CLSI method were determined also as non-susceptible by MBT ASTRA. However, 9 out of 33 strains that were categorized as susceptible by microdilution, were detected as non-susceptible by MBT ASTRA. A categorical agreement of 82% was obtained between the two methods, and sensitivity and specificity of 100% and 73% were calculated, respectively (Table 4) (Supplementary Table 1).

**Table 4:** Sensitivity, specificity, and categorical agreement of MBT ASTRA in comparison to CLSI microdilution results for anidulafungin, micafungin, and caspofungin.

**MBT ASTRA**	**Anidulafungin (%)**	**Micafungin (%)**	**Caspofungin (%)**
Sensitivity	100	100	100
Specificity	98	95.5	73
Categorical agreement	98	96	82

### MBT ASTRA on Positive Blood Culture

**Figure 3:**
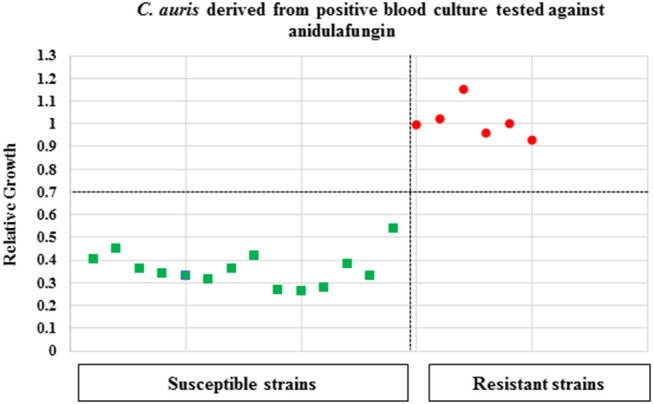
MBT ASTR4 of *C. auris* isolates derived from positive blood cultures. A total of 20 *C. auris* isolates (resistant = 6, susceptible = 14) derived from positive blood cultures were tested against anidulafungin. The MBT ASTRA breakpoint was determined at I μg/ml and an RG threshold of 0.7 for anidulafungin. All resistant and susceptible isolates were correctly detected.

The applicability of the MBT ASTRA for detecting yeast strains non-susceptible to echinocandins was directly investigated for cells derived from seeded blood cultures. Initially, spectra were analyzed by the MBT ASTRA prototype to determine the breakpoints and thresholds for the respective three antifungals. Afterwards, 14 susceptible and 6 non-susceptible strains (against anidulafungin and micafungin) were tested at the respective breakpoints and thresholds. The results for anidulafungin showed that the breakpoint was decreased to 1 μg/mL at a threshold of 0.7, compared to the results of cells cultured on SDA (Table 3). Beside this result, the breakpoint was reduced to 4 μg/mL for micafungin when an RG threshold of 0.7 was applied (Table 3). All 6 non-susceptible and 14 susceptible strains were correctly separated at these breakpoints of the corresponding antifungals and thresholds (Figure 3). For caspofungin, 6 out of 7 non-susceptible strains and twelve out of thirteen susceptible strains were correctly detected. In this case, the 2 strains that were misidentified, had been correctly detected by MBT ASTRA from colonies grown on SDA. These data demonstrate that the breakpoint for caspofungin has to be decreased to 1 μg/mL at a threshold of 0.7 (Table 3) when using MBT ASTRA.

## Discussion

This study investigated the identification of 50 *C. auris* isolates by MALDI-TOF MS, suggested CLSI microdilution cut-offs and evaluated MBT ASTRA for AFST on echinocandin. The results from this study demonstrate that a rapid MALDI-TOF MS technology not only can be used to unequivocally identify *C. auris*, but it has the potential to be successfully applied in rapid AFST. Recently, attention has focused on the identification of *C. auris* to distinguish it from close relatives like *C*. *haemulonii* using diverse biochemical-based testing, molecular, and commercial methods. A total of 50 *C. auris* isolates were correctly identified by MALDI-TOF MS with an accuracy of 100% when cultured on SDA and isolated from positive blood cultures. This was for a first time that applicability of MALDI-TOF MS was used to identify *C. auris* derived from positive blood cultures. This finding broadly supports the work of other studies in this area, demonstrating the high accuracy of MALDI-TOF MS for the identification of *C. auris* based on a well-established reference database, compared to other available methods. For instance, the results of this study are in agreement with those obtained by Bao JR *et al*., who more recently showed a high performance of the MALDI-TOF MS in identifying 23 clinical isolates of *C. auris* that were cultured on three different media. Their study indicated that the highest log scores were achieved for isolates grown on SDA (Bao et al., 2018).

Additionally, the present study demonstrates the successful applicability of MBT ASTRA for the detection of non-susceptible *C. auris* isolates to echinocandin drugs cultured on SDA. The most interesting finding was that 49 and 48 out of fifty *C. auris* isolates were correctly labeled as either susceptible or non-susceptible to anidulafungin and micafungin by MBT ASTRA, respectively. A sensitivity of 100% indicated that all 6 isolates non-susceptible against the respective antifungals were successfully detected. Notably, the same susceptible isolate was *mis*-identified as resistant for both antifungals even after repetition. The reason for this observation could be that this strain revealed an MIC in the microdilution that is next the breakpoint concentration. Considering that this strain had not been sequenced together with the allowed variance of the microdilution, it could be that this strain is indeed resistant. Further analysis is required to clarify this result.

To achieve valid results by MBT ASTRA, it is essential to first check the growth in the control setups. This was done by introducing a threshold for the minimum required growth calculated by the prototype software. Those strains with sufficient growth in the control setups were considered for further evaluation of sensitivity and specificity of the MBT ASTRA. Taken together, categorical agreement between MBT ASTRA and the CLSI was very high; 98% and 96% for anidulafungin and micafungin, respectively. Moreover, in the present study, the cut-offs found by the CLSI reference method for *C. auris* anidulafungin susceptibility testing confirmed the results obtained by other studies (Arendrup et al., 2017b; Chowdhary et al., 2018; Kordalewska et al., 2018; Spivak and Hanson, 2018), and the breakpoint found by MBT ASTRA was identical to the suggested (CDC) CLSI method. Importantly, the same breakpoint was obtained much more rapidly by MBT ASTRA, which requires up to 6 h, compared to 24/48 h used for the CLSI method in this study. For micafungin, the suggested cut-offs for the CLSI microdilution and MBT ASTRA were also identical, but these cut-offs were higher compared to those of other studies (Arendrup et al., 2017b; Chowdhary et al., 2018; Kordalewska et al., 2018). A recent study reported that micafungin to be the most potent echinocandin with an MIC approximately equal to that of anidulafungin (Kordalewska et al., 2018). In this study, the higher MICs obtained by the CLSI method and MBT ASTRA might be an epidemiological impact on fungal infections with a shift toward a reduced susceptibility of the strains against echinocandins (Arendrup and Patterson, 2017; Lockhart et al., 2017). Notably, results with caspofungin had lower specificity and sensitivity than anidulafungin or micafungin, with a categorical agreement between the CLSI microdilution and MBT ASTRA of 82%. Furthermore, the breakpoint found by MBT ASTRA was one serial dilution higher than that applied for the CLSI method. In accordance with these results, previous study reported challenges in identification of *FKS1* WT *C. auris* isolates for caspofungin antifungal susceptibility testing due to the presence of an ‘Eagle effect’ (Vanstraelen et al., 2013). This phenomenon has been notably reported for the caspofungin-caused reduction of the antifungal activity at high concentrations (Vanstraelen et al., 2013). As mentioned in the study by Espinel- Ingroff et al., variability of caspofungin MICs has been observed for *Candida spp*. used CLSI and EUCAST methods from different clinical laboratories. They have reported that most of the wild-type (WT) isolates (e.g., *C. glabrata* and *C. krusei*) were detected as either non-WT or resistant isolates (Espinel-Ingroff et al., 2013). Accordingly, Kordalewska et al. (2018) examined AFST of caspofungin for 106 *C. auris* isolates and found that only isolates with mutations in *FKS1* were resistant to echinocandins. Therefore, it is recommended that routine microdilution methods be avoided when detecting resistant strains without gene sequencing (Kordalewska et al., 2018). However, the reduced susceptibility of *C. auris* isolates to one or more echinocandin class drugs has been reported in several studies (Arendrup et al., 2017b; Kordalewska et al., 2018). A further extension of this MBT ASTRA investigation was to analyse its performance on positive blood culture samples. For anidulafungin and micafungin, all susceptible and non-susceptible strains were labeled correctly, with complete agreement between the CLSI method and MBT ASTRA. The breakpoint concentration was decreased by two and one serial dilution(s) for anidulafungin and micafungin, respectively. For caspofungin, one susceptible and one non-susceptible strain were *mis*-labeled resulting in a categorical agreement of 90%. Thus, for caspofungin, these results from blood culture bottles were more in agreement with CLSI method than those obtained from SDA.

Findings reported here shed new light on rapid and accurate antifungal susceptibility testing for echinocandin by MALDI-TOF MS. A new performance of MALDI-TOF MS for rapid AFST based on changes in the profile spectra in the presence of antifungals was recently introduced by Vella et al. (2013), but however, they revealed that this approach could not perfectly be applied for *C. glabrata* strains resistant to anidulafungin with known *FKS2* mutations (Vella et al., 2017). In this study, the CLSI method was considered as a gold standard, however, it is not a perfect method and some susceptible isolates are not clearly detected by the CLSI method. Hence, the MBT ASTRA might be even more helpful in the future. In summary, MBT ASTRA has been shown to be applicable for rapid AFST in *C. albicans, C. glabrata* (Vatanshenassan et al., 2018), and *C. auris*. Applicability for further yeast species and antifungals will be necessary.

## Ethics Statement

According to common sense, for this study no ethical approval from an ethics committee is required, because all strains were derived either from strain collections or stock cultures of routine samples. The isolates used in this non-clinical *in vitro* study were obtained during routine patient care for which no written informed consent is required. The local Institutional Review Board of the hospital determined that ethics approval and consent from admitted patients was not required according to national and institutional guidelines.

## Author Contributions

MV contributed to the conceptualization, the investigation, the methodology, and the writing of the original draft. MK contributed to the conceptualization the methodology, the scientific advice, the project administration, the review, and editing of the manuscript, and the funding acquisition. KS contributed to the conceptualization, the methodology, the scientific advice, the project administration, and the review and editing of the manuscript. TB reviewed and edited the manuscript. JM reviewed and edited the manuscript, contributed to providing a sample, and scientific advice. JB and AC reviewed and edited the manuscript, and provided a sample. RB-A provided a sample.

### Conflict of Interest Statement

MK, KS, and MV are employees of the mass spectrometry company Bruker Daltonik GmbH. The remaining authors declare that the research was conducted in the absence of any commercial or financial relationships that could be construed as a potential conflict of interest.
