# Supplementary material for: Candida auris Identification and Rapid Antifungal Susceptibility Testing Against Echinocandins by MALDI-TOF MS
Source: Front Cell Infect Microbiol. 2019 Feb 18;9:20. doi: 10.3389/fcimb.2019.00020 (PMC6387932; doi:10.3389/fcimb.2019.00020)
Supplement: Supplementary file 1 [file Data_Sheet_1.PDF]

Supplementary Material

Table 1. Overview of the 50 *C. auris* strains used in this study. In vitro anidulafungin, micafungin and caspofungin susceptibility test using CLSI and MBT ASTRA for respective isolates have been shown. Six strains were constantly resistant against 3 antifungals by 2 different methods (strains number 22, 24-28).

	***C. auris* Strains**	**Origin**	**CLSI MIC (Visually) [μg/ml] 24 h**			**MBT ASTRA MIC [μg/ml] 24 h**			**Amino acid substitution in FKS1 HSI**
			**Anidulafungin**	**Micafungin**	**Caspofungin**	**Anidulafungin**	**Micafungin**	**Caspofungin**	
**1**	CDC_381 [ac]	Japan	0.5	4	0.25	0.125	4	1	Not available
**2**	CDC_382 [ac]	Unknown	1	4	0.25	0.125	4	1	Not available
**3**	CDC_383 [ac]	South Africa	1	4	0.5	0.25	2	1	Not available
**4**	CDC_384 [ac]	South Africa	1	4	0.25	0.125	1	0.5	Not available
**5**	CDC_385 [ac]	Venezuela	1	4	0.5	0.25	4	0.5	Not available
**6**	CDC_386 [ab]	Venezuela	1	4	0.25	0.5	4	2	Not available
**7**	CDC_387 [ac]	Pakistan	1	8	0.25	2	4	2	Not available
**8**	CDC_388 [ac]	Pakistan	4	4	0.5	2	8	>8	Not available
**9**	CDC_389 [ac]	India	0.5	4	0.25	4	>8	8	Not available
**10**	CDC_390 [ab]	India	2	8	>16	0.125	1	8	Not available
**11**	TAU_171103-23 [ab]	Israel	0.5	8	0.5	0.125	4	0.5	No mutations
**12**	TAU_171103-24 [ab]	Israel	0.5	8	0.5	0.125	1	0.5	No mutations
**13**	TAU_171103-156 [ab]	Israel	0.5	8	0.5	0.25	8	1	No mutations
**14**	TAU_171103-172 [ab]	Israel	0.5	8	0.5	0.125	4	1	No mutations
**15**	TAU_171103-197 [ab]	Israel	0.5	8	0.5	0.125	0.5	1	No mutations
**16**	TAU_171103-201 [ab]	Israel	0.5	8	0.5	2	4	1	No mutations
**17**	TAU_171103-597 [ab]	South Africa	1	8	0.5	0.125	1	0.5	No mutations
**18**	TAU_171103-598 [ab]	South Africa	1	8	0.5	0.5	2	1	No mutations
**19**	CBS 31062 [ab]	India	0.25	4	0.25	0.06	4	0.125	No mutations
**20**	KCTC 17810 [ab]	India	0.25	4	0.125	0.5	4	1	No mutations
**21**	CWZ_10031064 [ab]	India	0.25	4	0.25	0.125	2	0.125	No mutations
**22**	CWZ_10051257 [ab]	India	>16	>16	>16	>8	>8	8	S639F
**23**	CWZ_10051259 [ab]	India	2	8	1	0.06	8	8	No mutations
**24**	CWZ_10051262 [ab]	India	>16	>16	>16	>8	>8	8	S639F
**25**	CWZ_10051266 [ab]	India	>16	>16	>16	>8	>8	8	S639F
**26**	CWZ_10051295 [ab]	India	>16	>16	>16	>8	>8	8	S639F
**27**	CWZ_10051297 [ab]	India	>16	>16	>16	>8	>8	8	S639F
**28**	Bgm_180321 [ac]	Kuwait	16	16	>16	>8	>8	>8	Not available

**29**	CBS 10913 [ab]	Japan	0.5	2	0.25	0.125	2	1	Not available
**30**	CBS 12372 [ab]	Korea	2	4	0.25	0.125	0.5	0.125	Not available
**31**	CBS 12766 [ab]	India	2	8	1	2	1	>8	Not available
**32**	CBS 12767 [ab]	India	2	8	0.5	0.25	2	1	Not available
**33**	CBS 12768 [ab]	India	2	4	0.5	0.25	2	2	Not available
**34**	CBS 12769 [ab]	India	2	4	0.5	2	2	1	Not available
**35**	CBS 12770 [ab]	India	2	2	>16	1	8	>8	Not available
**36**	CBS 12771 [ab]	India	2	8	0.5	2	4	8	Not available
**37**	CBS 12772 [ab]	India	2	4	>16	2	2	>8	Not available
**38**	CBS 12773 [ab]	India	2	4	0.5	2	4	8	Not available
**39**	CBS 12774 [ab]	India	0.25	4	1	0.06	4	>8	Not available
**40**	CBS 12775 [ab]	India	0.125	0.125	0.06	0.5	2	>8	Not available
**41**	CBS 12776 [ab]	India	4	8	>16	1	4	>8	Not available
**42**	CBS 12777 [ab]	India	1	8	>16	0.5	2	>8	Not available
**43**	CBS 12805 [ab]	India	2	8	1	0.5	4	>8	Not available
**44**	CBS 12806 [ab]	India	2	4	0.5	0.5	2	>8	Not available
**45**	CBS 12876 [ab]	India	0.25	2	0.5	2	4	>8	Not available
**46**	CBS 12877 [ab]	India	4	8	>16	2	4	>8	Not available
**47**	CBS 14916 [ab]	Oman	0.5	4	0.5	2	4	>8	Not available
**48**	CBS 14918 [ab]	Oman	2	8	1	>8	>8	>8	Not available
**49**	CBS 15108 [ab]	Oman	0.25	2	0.25	0.25	4	0.5	Not available
**50**	CBS 15109 [ab]	Oman	0.25	2	0.25	0.25	2	>8	Not available
	ATCC 64548 [a]	Quality control	0.06	0.125	0.06	≤0.25	≤0.5	≤0.5	
	ATCC 64550 [a]	Quality control	0.06	0.06	0.125	≤0.25	≤0.5	≤0.25	
	ATCC 22019 [×a]	Quality control	1	2	2	×	×	×	
	ATCC 6258 [×a]	Quality control	0.125	0.25	0.25	×	×	×	

× These quality control strains were only tested by CLSI.

a. These strains were identified by MALDI TOF MS.

b. These strains were identified by Molecular methods.

c. Information about identification based molecular method is not available.
